# Utility of RGNEF in the Prediction of Clinical Prognosis in Patients with Rectal Cancer Receiving Preoperative Concurrent Chemoradiotherapy

**DOI:** 10.3390/life12010018

**Published:** 2021-12-23

**Authors:** Chih-I. Chen, Hsin-Pao Chen, Kuang-Wen Liu, Chu-Chun Chien, Yu-Ching Wei

**Affiliations:** 1 Division of Colon and Rectal Surgery, Department of Surgery, E-DA Hospital, Kaohsiung 824, Taiwan; jimmyee0901@gmail.com (C.-I.C.); ed102430@edah.org.tw (H.-P.C.); ed100739@edah.org.tw (K.-W.L.); 2 Division of General Medicine Surgery, Department of Surgery, E-DA Hospital, Kaohsiung 824, Taiwan; 3 School of Medicine, College of Medicine, I-Shou University, Kaohsiung 840, Taiwan; 4 Department of Information Engineering, I-Shou University, Kaohsiung 840, Taiwan; 5 The School of Chinese Medicine for Post Baccalaureate, I-Shou University, Kaohsiung 840, Taiwan; 6 Department of Pathology, School of Medicine, College of Medicine, Kaohsiung Medical University, Kaohsiung 807, Taiwan; 1060566@kmuh.org.tw; 7 Department of Pathology, Kaohsiung Municipal Ta-Tung Hospital, Kaohsiung 801, Taiwan

**Keywords:** ARHGEF28, RGNEF, rectal cancer

## Abstract

Rectal cancer is a heterogeneous malignancy with different clinical responses to preoperative concurrent chemoradiotherapy (CCRT). To discover the significant genes associated with CCRT response, we performed data mining of a transcriptomic dataset (GSE35452), including 46 rectal cancer patients who received preoperative CCRT and underwent standardized curative resection. We identified ARHGEF28 as the most significantly upregulated gene correlated with resistance to CCRT among the genes related to Rho guanyl-nucleotide exchange factor activity (GO:0005085). We enrolled 172 patients with rectal cancer receiving CCRT with radical surgery. The expression of ARHGEF28 encoded protein, Rho guanine nucleotide exchange factor (RGNEF), was assessed using immunohistochemistry. The results showed that upregulated RGNEF immunoexpression was considerably correlated with poor response to CCRT (*p* = 0.018), pre-CCRT positive nodal status (*p* = 0.004), and vascular invasion (*p* < 0.001). Furthermore, high RGNEF expression was significantly associated with worse local recurrence-free survival (*p* < 0.0001), metastasis-free survival (MeFS) (*p* = 0.0029), and disease-specific survival (DSS) (*p* < 0.0001). The multivariate analysis demonstrated that RGNEF immunoexpression status was an independent predictor of DSS (*p* < 0.001) and MeFS (*p* < 0.001). Using Gene Ontology enrichment analysis, we discovered that ARHGEF28 overexpression might be linked to Wnt/β-catenin signaling in rectal cancer progression. In conclusion, high RGNEF expression was related to unfavorable pathological characteristics and independently predicted worse clinical prognosis in patients with rectal cancer undergoing CCRT, suggesting its role in risk stratification and clinical decision making.

## 1. Introduction

Colorectal cancer (CRC) is heterogeneous and constitutes 10% of all cancer diagnoses. It is the third most prevalent cancer and the second leading cause (9.8%) of cancer death worldwide [1]. Rectal cancer, developing within 15 cm of the anal verge, accounts for about 40% of CRC cases and is associated with worse clinical outcomes [1,2]. According to the GLOBOCAN 2020 data, there were an estimated 732,210 new cases of rectal cancer and 339,022 deaths [1].

Locally advanced rectal cancer (LARC) remains a challenging malignancy. A multidisciplinary approach combining chemotherapy, radiotherapy, and surgery is required for optimal outcomes. Fluoropyrimidine-based neoadjuvant concurrent chemoradiotherapy (CCRT) reduces the risk of local recurrence in patients with LARC [3,4,5]. However, metastases rates of 20–30% are still reported, and patient survival rates have not significantly improved, despite curative surgery [3,4,5]. Therefore, new predictive biomarkers are needed for better patient stratification to ensure appropriate therapy.

During cancer progression and metastasis, tumor cells experience an epithelial-to-mesenchymal transition associated with increased cell motility [6]. Cell migration plays a vital role in cancer local invasion and distant metastasis. This involves the dissolution of cell-cell contacts and the development of integrin containing cell-substratum structures, termed focal adhesions. The migration cycle is partly regulated by Rho-family GTPase activity [7].

Rho-family GTPases, comprising Rac, Rho, and Cdc42, are key effectors of actin cytoskeletal dynamics, membrane protrusion, and cell migration. They play the roles of molecular switches, cycling between an inactive guanosine diphosphate (GDP)-bound form and an active guanosine triphosphate (GTP)-bound state that interacts with effector targets [7,8]. Some evidence suggests that activation of Rho GTPases is critical for tumor invasion and nodal and distant metastasis in CRC [9,10,11]. Rho GTPases are activated by guanine nucleotide exchange factors (GEFs) that catalyze the exchange of GDP for GTP [7,8]. However, the genes associated with Rho guanyl-nucleotide exchange factor activity in rectal cancer remain to be identified.

We performed expression profiling of a transcriptomic dataset to discover differentially expressed genes (DEGs) associated with Rho guanyl-nucleotide exchange factor activity (GO:0005085) in rectal cancer progression after CCRT. The results showed that ARHGEF28 was the most significantly upregulated gene involved in CCRT resistance in rectal cancer. Human ARHGEF28 is located on the long arm of chromosome 5 (5q13.2). It encodes a 190 kDa protein, Rho guanine nucleotide exchange factor (RGNEF), which is a member of the diffuse B-cell lymphoma (Dbl) family of GEFs [12,13].

High RGNEF expression promoted colon cancer cell motility, invasion, and tumor progression [14,15,16]. Elevated ARHGEF28 mRNA levels have been associated with late-stage serous ovarian cancer and worse clinical outcomes [17]. RGNEF is the only member that combines GEF activity with RNA-binding activity. It regulates RNA metabolism in motor neurons, which is important in the pathogenesis of amyotrophic lateral sclerosis (ALS), a motor neuron disease [18]. It also possesses a prosurvival effect, anti-apoptotic activity, dendritic morphogenesis, and synapse formation [19,20,21,22]. However, the clinical relevance of RGNEF in rectal cancer has not been elucidated. Therefore, we wanted to clarify the role of RGNEF in preoperative CCRT response and compare RGNEF immunoexpression with patient outcomes and clinicopathological features in our rectal cancer cohort.

## 2. Materials and Methods

### 2.1. Data Mining of a Rectal Cancer Microarray Dataset

We explored the DEGs correlated to CCRT response in a gene expression omnibus (GEO) dataset (GSE35452), including 46 patients with rectal cancer. Before starting preoperative CCRT, biopsy specimens were collected during colonoscopy and analyzed using the Affymetrix Human Genome U133 Plus 2.0 Array [23]. To computerize the gene expression levels, we imported raw CEL files into the Nexus Expression 3 software (BioDiscovery, El Segundo, CA, USA). According to the clinical response to CCRT, we compared “responders” and “nonresponders” to identify significant DEGs related to Rho guanyl-nucleotide exchange factor activity (GO:0005085). DEGs with *p* < 0.01 and log2 ratio > 0.15 were chosen for further evaluation.

### 2.2. Patients and Samples

We enrolled 172 consecutive patients with rectal cancer who received CCRT, followed by total mesorectal excision (anterior resection or abdominoperineal resection) between 1998 and 2004. Colonoscopic biopsy was performed to confirm the diagnosis of rectal adenocarcinoma. None of the patients had distant metastasis on chest radiography and abdominopelvic computed tomography. All patients received preoperative CCRT, including radiotherapy and concomitant 5-fluorouracil-based chemotherapy, with a total dose of 45–50 Gy in 25 fractions over 5 weeks before total mesorectal excision, as described previously [24]. Patients with pre-CCRT or post-CCRT T3–T4 staging or nodal metastatic disease received adjuvant chemotherapy. Patient features, pathological characteristics, and clinical outcomes were retrospectively reviewed. The study protocol was approved by the ethics committee (IRB10302014).

### 2.3. Histopathological Assessments and Immunohistochemical Scoring

Two independent pathologists reviewed all tumor specimens without knowledge of the patients’ clinical information. We determined tumor stages according to the seventh edition of the AJCC/UICC tumor-node-metastasis staging system. Tumor regression grade after CCRT was evaluated based on the Dworak grading system [25]. Immunohistochemical (IHC) staining was performed following standard protocols as described in our previous study [24]. We incubated the tissues with an anti-RGNEF monoclonal antibody (GTX54661, GeneTex). Two independent pathologists evaluated the intensity and percentage of positive staining cancer cells to yield the H-score, using the following equation: ΣPi(i + 1), where Pi is the percentage (0–100%) of stained tumor cells for each intensity, and i is the intensity of staining (0–3+). We used the median H-score to divide immunoreactivity into high and low expression levels.

### 2.4. Gene Ontology Enrichment Analysis

To estimate the ARHGEF28 gene functions in rectal cancer, we investigated the correlations between ARHGEF28 and its co-expressed genes in the colorectal adenocarcinoma dataset from The Cancer Genome Atlas (TCGA) database (http://cbioportal.org, accessed on 1 February 2021). The top 200 transcripts showing negative or positive correlations with ARHGEF28 were further examined using the Gene Ontology (GO) classification system (http://geneontology.org/, accessed on 8 February 2021), based on three functional groups: biological processes, molecular functions, or cellular components.

### 2.5. Statistical Analysis

Pearson’s chi-square test was used to evaluate the correlations of the RGNEF expression status with different clinicopathological features. The Kaplan–Meier method with the log-rank test was applied to estimate the effect of the RGNEF expression status on patient outcomes, including disease-specific survival (DSS), local recurrence-free survival (LRFS), and metastasis-free survival (MeFS) measured from the date of operation to the time of cancer death, metastatic development, and local tumor recurrence. To evaluate the independent predictors of DSS, LRFS, and MeFS, univariate and multivariate analyses were performed using Cox proportional hazards regression model. Two-tailed analyses with *p*-values <0.05 were considered significant. Statistical analysis was performed using the SPSS software (IBM, Armonk, NY, USA).

## 3. Results

### 3.1. ARHGEF28 Is the Most Upregulated DEG Related to CCRT Resistance in Rectal Cancer Associated with Rho Guanyl-Nucleotide Exchange Factor Activity

**Figure 1:**
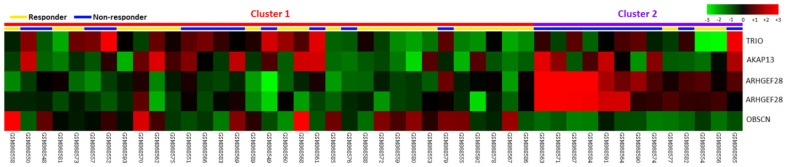
Expression profiling of genes associated with Rho guanyl-nucleotideexchange factor activity (GO:0005085) in relation to the response to concurrent chemoradiotherapy (CCRT). We identified ARHGEF28 as the most significantly upregulated gene related to CCRT resistance.

Data mining of a GEO dataset (GSE35452) was performed, comprising of 46 patients receiving preoperative CCRT; 22 patients (47.8%) were categorized as non-responders and 24 patients (52.2%) were classified as responders to identify the genetic biomarkers to predict the preoperative CCRT response in rectal cancer patients. Focusing on Rho guanyl-nucleotide exchange factor activity (GO:0005085), we identified five probes covering four genes: ARHGEF28, TRIO, AKAP13, and OBSCN (Table 1 and Figure 1) related to CCRT response. ARHGEF28 was selected for further analysis because it was the most upregulated gene related to CCRT resistance. Furthermore, the clinical relevance of ARHGEF28 in rectal cancer is not well understood. We further studied the correlations between the ARHGEF28 encoded protein, RGNEF expression levels, and clinicopathological characteristics and their prognostic impacts in our cohort.

### 3.2. Clinicopathological Features

The characteristics of our cohort are listed in Table 2. We included 172 patients with rectal cancer, including 64 (37.2%) females and 108 (62.8%) males. Moreover, 91 (52.9%) patients had advanced rectal cancer (cT3-T4), and 47 (27.3%) patients had lymph node metastatic disease at the time of initial cancer diagnosis. After CCRT, lymph node metastasis (ypN1-2) was confirmed in 49 (28.5%) patients, and tumor invasion beyond the muscularis propria (ypT3-4) was noted in 86 (50%) patients. Perineural invasion and vascular invasion were detected in five (2.9%) and 15 (8.7%) patients, respectively. Regarding tumor response to CCRT, we found that 17 patients (9.9%) had a complete response (grade 4), 118 patients (68.6%) had a modest response (grade 2–3), and 37 patients (21.5%) had little or no response (grade 0–1).

### 3.3. Association between RGNEF Expression and Clinicopathological Parameters

**Figure 2:**
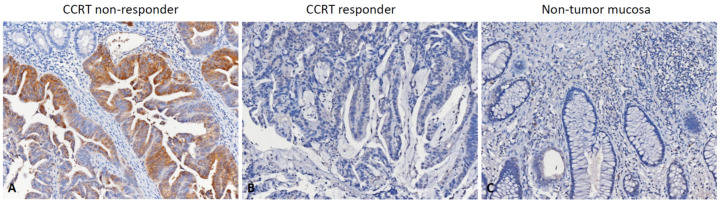
Immunohistochemical expression of Rho guanine nucleotide exchange factor (RGNEF): (**A**) representative images of rectal cancer exhibiting high RGNEF immunoexpression among concurrent chemoradiotherapy (CCRT) non-responders; (**B**) low RGNEF immunoexpression among CCRT responders; (**C**) no RGNEF immunoexpression in non-tumor mucosa.

We used IHC staining to assess the RGNEF expression level (Figure 2) and correlated its immunoexpression with clinicopathological characteristics in our cohort to verify the association between RGNEF and rectal cancer. High RGNEF immunoexpression was significantly associated with high pre-CCRT nodal metastasis (*p* = 0.004) and vascular invasion (*p* < 0.001). Additionally, patients with high RGNEF expression had a lower complete tumor regression (grade 4) rate than those with low RGNEF expression (4.7% vs. 15.1%, *p* = 0.018).

### 3.4. Survival and Prognostic Significances of RGNEF Expression

**Figure 3:**
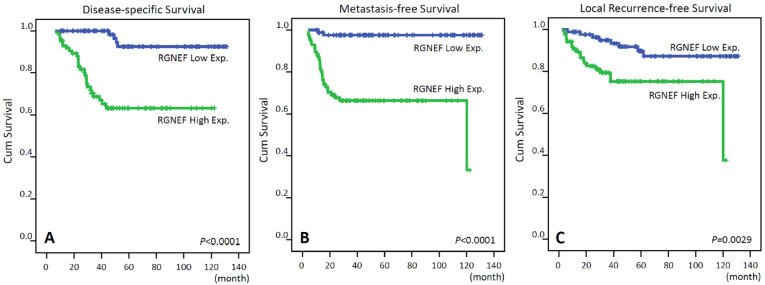
Kaplan–Meier survival analysis showing that high Rho guanine nucleotide exchange factor (RGNEF). Immunoexpression was significantly correlated with worse: (**A**) disease-specific survival; (**B**) metastasis-free survival.; (**C**) local recurrence-free survival.

The mean follow-up period was 30.8 months. Thirty-one (18.0%) deaths occurred due to cancer progression. Moreover, local tumor recurrence and distant metastasis subsequently developed in 27 (15.7%) and 31(18.0%) patients, respectively. We then did univariate and multivariate analyses to evaluate the predictors of DSS, LRFS, and MeFS. High RGNEF expression levels were associated with cancer deaths (31.4% vs. 4.7%), postoperative distant metastasis (33.7% vs. 2.3%), and postoperative local tumor recurrence (22.1% vs. 9.3%). Remarkably, the results of univariate analysis demonstrated that high RGNEF immunoexpression (Figure 3), pre-CCRT lymph node metastasis, and high post-CCRT tumor stage were significantly associated with worse DSS, LRFS, and MeFS. In addition, vascular invasion, and low tumor regression rate showed the same results in the univariate analysis (Table 3). Moreover, the multivariate analysis showed that high RGNEF immunoexpression was an independent predictor of metastasis occurrence (hazard ratio (HR), 17.011; 95% confidence interval (CI), 3.986–72.602; *p* < 0.001) and cancer-related death (HR, 7.985; 95% CI, 2.708–23.547; *p* < 0.001) (Table 4). For local tumor recurrence, tumor regression grade (*p* = 0.044) was a significant predictor in the multivariate analysis.

### 3.5. ARHGEF28 Overexpression May Be Linked to Wnt/β-Catenin Signaling in Rectal Cancer

**Figure 4:**
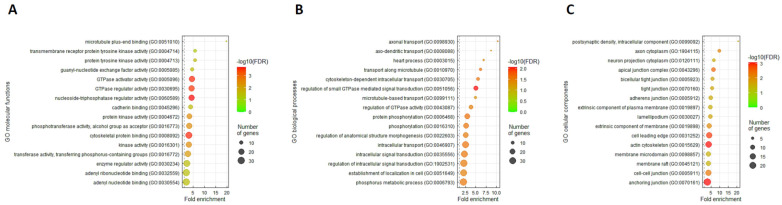
Gene Ontology enrichment analysis on ARHGEF28 in different functional groups: (**A**) molecular functions; (**B**) biological processes; (**C**) cellular components.

**Figure 5:**
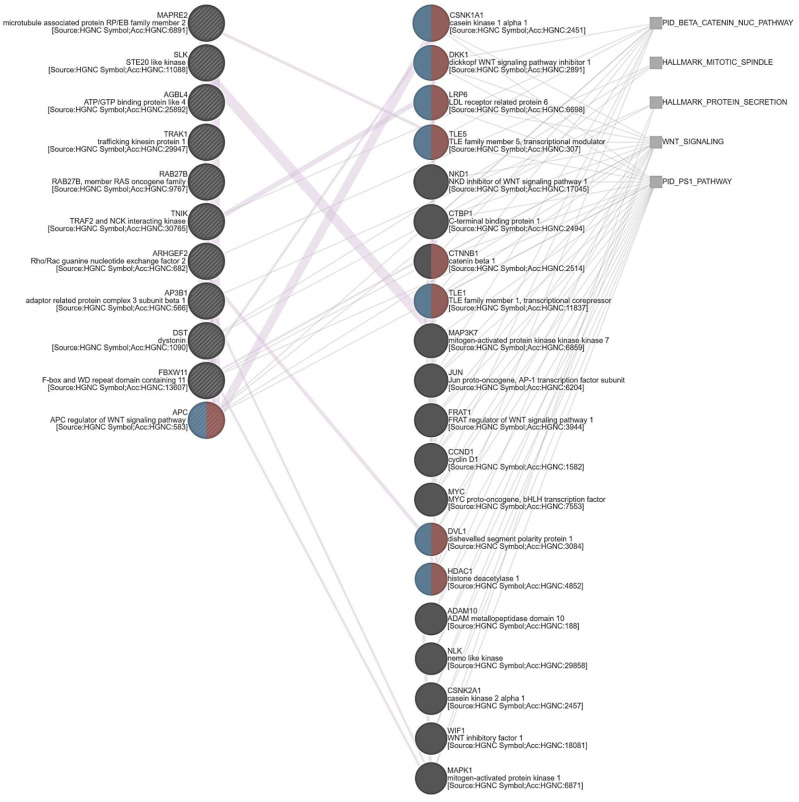
Annotated by the Molecular Signatures Database, we identified several distinguished pathways, including the Wnt and nuclear β-catenin signaling and presenilin 1 pathways.

We conducted a gene co-expression analysis to speculate ARHGEF28 function in rectal cancer. Applying the colorectal adenocarcinoma dataset from TCGA (*n* = 594), we appraised the top 200 differentially expressed genes that showed positive (Table S1) or negative (Table S2) correlations with ARHGEF28. The GO classification system was then used for functional annotation. In terms of molecular functions, we recognized microtubule plus-end binding (GO: 0051010, fold enrichment: 19.71) as the most significant GO term positively correlated with ARHGEF28 (Figure 4A). Interestingly, we also identified axonal transport (GO: 0098930, fold enrichment: 10.35) and postsynaptic density and intracellular components (GO: 0099092, fold enrichment: 20.7) as the most remarkable GO terms correlated with ARHGEF28 upregulation in the context of biological processes and cellular components, respectively (Figure 4B,C). Furthermore, utilizing the GeneMANIA prediction server [26], a weighted network was constructed to connect critical genes that were implicated in the GO terms mentioned above to each other. These genes included microtubule-associated protein RP/EB family member 2, dystonin, adenomatous polyposis coli (APC), F-box and WD repeat domain-containing 11, Ras-related protein Rab-27B, and adaptor-related protein complex 3 subunit beta 1. In addition, genes such as AGBL carboxypeptidase 4, trafficking kinesin protein 1, tumor necrosis factor receptor-associated factor 2 and NCK-interacting kinase, STE20-like kinase, and Rho/Rac guanine nucleotide exchange factor 2 were also studied. Annotated by the Molecular Signatures Database, we identified several distinct pathways, including Wnt and nuclear beta-catenin (β-catenin) signaling and presenilin 1 (PSEN1, also known as PS1) (Figure 5). We performed immunostaining of RGNEF and β-catenin, and demonstrated the correlation of these protein expressions (Figure S1). It has been well recognized that mutant PSEN1 (gain of function) is associated with the accumulation of amyloid beta (Aβ) and the onset of Alzheimer’s disease [27]. Altogether, these findings suggest that ARHGEF28 may promote rectal cancer progression through neurodegeneration-related genes and the Wnt/β-catenin signaling pathway.

## 4. Discussion

Preoperative CCRT is the standard treatment for patients with LARC before surgical resection or for unresectable LARC. This treatment can increase tumor resectability, downstage tumors, and lower the possibility of positive surgical margins and local recurrence. However, only 10–20% of patients can achieve a complete pathological response after undergoing CCRT. The 5-year disease-free survival rate is approximately 70%, and 15–20% of patients develop local recurrence or distant metastasis [28]. These results encouraged us to study valuable predictive and prognostic markers for better patient stratification. In this study, we demonstrated that high RGNEF immunoexpression is associated with a low CCRT response rate and aggressive cancer features. Patients with high RGNEF expression had an increased risk of cancer death, local tumor recurrence, and metastatic development.

RGNEF is a member of the Dbl family of GEFs [12]. This family can activate Rho GTPase functions and react to several effector targets, resulting in diverse cellular responses, for example endocytosis, exocytosis, cytoskeleton reorganization, stimulation of DNA synthesis, and transcription activation [12]. RGNEF is unique in the human proteome because it can regulate GEF activity and mRNA stability [29]. It also serves as a stress response factor and protects cells from oxidative or osmotic stress [30]. RGNEF has been demonstrated to play a critical role in ALS forming neuronal cytoplasmic inclusions that co-aggregate with other ALS-related proteins in spinal cord motor neurons [31].

Additionally, RGNEF plays a role in colon cancer and serous ovarian cancer. In patients with colon cancer, elevated ARHGEF28 mRNA expression was found in advanced-stage disease [16]. Yu et al. discovered that RGNEF interaction with focal adhesion kinase (FAK) facilitated cell motility, invasiveness, and invadopodia formation in colon DLD-1 cancer cells [16,32]. The RGNEF-FAK signaling complex promotes tumor growth and local invasion of orthotopic colon cancer in mice [32]. Recently, increased RGNEF mRNA and protein expression were reported in advanced serous ovarian cancer, which were associated with worse patient overall and progression-free survival rates [17]. Knockout of RGNEF inhibited primary and metastatic ovarian tumor formation, decreased antioxidant gene signature, and elevated cellular reactive oxygen species, thus lowering spheroid formation and promoting anoikis. RGNEF re-expression facilitates nuclear factor-κB-dependent tumor sphere survival [17].

Aberrant activation of Wnt/β-catenin signaling is involved in diverse cancers, especially in the initiation and progression of CRC [33]. Impressively, the gene co-regulation network correlated with ARHGEF28 upregulation showed that many genes were implicated in the Wnt/β-catenin pathway (Figure 5). Positively correlated with ARHGEF28, APC (Spearman’s correlation: 0.392) was identified as one of the key genes involved in this pathway (Figure S2). APC was originally considered to target β-catenin for degradation and act as a tumor suppressor. Nevertheless, following Wnt exposure, APC has recently been suggested to recruit the β-catenin destruction complex to the cell membrane to release undegraded β-catenin in human colon epithelial cells [34]. In addition to regulating CRC development, Wnt/β-catenin signaling has also been demonstrated to confer chemoradiotherapy resistance in rectal cancer [35]. On the other hand, the PS1 pathway was also significantly involved in the gene co-regulation network correlated with ARHGEF28 upregulation. ARHGEF28 has been linked to neurodegeneration and cell survival [18]. Additionally, PS1, a part of the gamma-(γ-)secretase complex, is regarded to cleave amyloid precursor protein and generate Aβ, and is associated with the onset of Alzheimer’s disease. Employing the GEPIA database (http://gepia.cancer-pku.cn/detail.php?gene=PSEN1, accessed on 1 September 2021), we discovered that the PS1 transcripts increased in rectal adenocarcinoma (*n* = 152) in comparison with the paired normal rectal tissues (*n* = 92). Moreover, PS1 has been reported to cleave E-cadherin and release its bound partner β-catenin, translocating from the cell membrane to the nucleus. Then it triggers gastric cancer progression by activating Wnt target gene transcription [36]. Accordingly, the correlations among the expression of ARHGEF28, the PS1 and Wnt/β-catenin pathways, and rectal cancer development and CCRT resistance require further investigation.

Some limitations of our study should be addressed. First, this was a retrospective study, and all patients were from a single institution. Second, we did not investigate ARHGEF28 mRNA level in rectal cancer, which may further support our findings. Third, we have explored the possible molecular mechanism of RGNEF in cancer progression using bioinformatics analysis, but in vitro or in vivo experiments are warranted to elucidate these results. Finally, the number of samples was not sufficient. The predictive impact of RGNEF immnoexpression in rectal cancer should be verified by large prospective multicenter studies.

## 5. Conclusions

Our results demonstrated that RGNEF expression was an independent predictor of clinical outcomes for patients with rectal cancer receiving CCRT. High RGNEF expression is associated with aggressive cancer features. Integrating RGNEF immunostaining with standard prognostic factors can help physicians stratify patient risk. Elucidating the molecular mechanisms and biological pathways of RGNEF in rectal cancer progression may lead to new therapeutic targets.

## Figures and Tables

**Table 1:** Summary of differentially expressed genes associated with Rho guanyl-nucleotide exchange factor activity (GO:0005085) in relation to response to CCRT in rectal cancer.

Probe	Comparison Log Ratio	Comparison *p*-Value	Gene Symbol	Gene Name	Biological Process	Molecular Function
1554004_a_at	0.4167	0.0028	*ARHGEF28*	Rho-guanine nucleotide exchange factor 28	Intracellular signaling cascade, regulation of Rho protein signal transduction	Rho guanyl-nucleotide exchange factor activity
216697_at	0.2424	0.0002	*TRIO*	Triple functional domain (PTPRF interacting)	protein amino acid phosphorylation, regulation of Rho protein signal transduction, transmembrane receptor protein tyrosine phosphatase signaling pathway	ATP binding, Rho guanyl-nucleotide exchange factor activity, guanyl-nucleotide exchange factor activity, kinase activity, nucleotide binding, protein kinase activity, protein serine/threonine kinase activity, transferase activity
222023_at	0.1782	0.0099	*AKAP13*	A kinase (PRKA) anchor protein 13	Intracellular signaling cascade, regulation of Rho protein signal transduction	Rho guanyl-nucleotide exchange factor activity, diacylglycerol binding, guanyl-nucleotide exchange factor activity, metal ion binding, receptor activity, signal transducer activity, zinc ion binding
232994_s_at	0.4723	0.0037	*ARHGEF28*	Rho-guanine nucleotide exchange factor 28	Intracellular signaling cascade, regulation of Rho protein signal transduction	Rho guanyl-nucleotide exchange factor activity
233029_at	−0.3843	0.0097	*OBSCN*	Obscurin; cytoskeletal calmodulin and titin-interacting RhoGEF	Cell differentiation, multicellular organismal development, protein amino acid phosphorylation, regulation of Rho protein signal transduction	ATP binding, Rho guanyl-nucleotide exchange factor activity, kinase activity, magnesium ion binding, metal ion binding, nucleotide binding, protein binding, protein kinase activity, protein serine/threonine kinase activity, protein-tyrosine kinase activity, transferase activity

**Table 2:** Associations and comparisons between RGNEF expression and clinicopathological factors.

Parameter		No.	RGNEF Expression		*p*-Value
			Low Exp	High Exp.	
Gender	Male	108	50	58	0.207
	Female	64	36	28	
Age	<70	106	49	57	0.210
	≥70	66	37	29	
Pre-Tx Tumor Status (cT)	T1-T2	81	43	38	0.445
	T3-T4	91	43	48	
Pre-Tx Nodal Status (cN)	N0	125	71	54	0.004 *
	N1-N2	47	15	32	
Post-Tx Tumor Status (ypT)	T1-T2	86	46	40	0.360
	T3-T4	86	40	46	
Post-Tx Nodal Status (ypN)	N0	123	61	62	0.866
	N1-N2	49	25	24	
Vascular Invasion	Absent	157	85	72	<0.001 *
	Present	15	1	14	
Perineural Invasion	Absent	167	84	83	0.650
	Present	5	2	3	
Tumor Regression Grade	Grade 0–1	37	13	24	0.018 *
	Grade 2–3	118	60	58	
	Grade 4	17	13	4	

* statistically significant.

**Table 3:** Univariate log-rank analysis for important clinicopathological variables and RGNEF expression.

Parameter		No. of Case	DSS		LRFS		MeFS	
			No. of Event	*p*-Value	No. of Event	*p*-Value	No. of Event	*p*-Value
Gender	Male	108	20	0.9026	7	0.2250	17	0.3520
	Female	64	11		20		14	
Age	<70	106	19	0.8540	18	0.6615	20	0.7427
	≥70	66	12		9		11	
Pre-Tx tumor status (cT)	T1-T2	81	10	0.0776	10	0.2261	11	0.1745
	T3-T4	91	21		17		20	
Pre-Tx nodal status (cN)	N0	125	19	0.0711	15	0.0070 *	19	0.0973
	N1-N2	47	21		12		12	
Post-Tx tumor status (ypT)	T1-T2	86	7	0.0006 *	7	0.0040 *	8	0.0033 *
	T3-T4	86	24		20		23	
Post-Tx nodal status (ypN)	N0	123	21	0.5998	16	0.1320	20	0.4634
	N1-N2	49	10		11		11	
Vascular invasion	Absent	157	25	0.0184 *	21	0.0028 *	27	0.4470
	Present	15	6		6		4	
Perineural invasion	Absent	167	29	0.2559	25	0.0940	30	0.9083
	Present	5	2		2		1	
Tumor regression grade	Grade 0–1	37	13	0.0038 *	10	0.0090 *	14	0.0006 *
	Grade 2–3	118	17		17		16	
	Grade 4	17	1		0		1	
Down stage after CCRT	Non-Sig.	150	29	0.1651	24	0.5961	30	0.0853
	Sig. (≥2)	22	2		3		1	
RGNEF expression	Low Exp.	86	4	<0.0001 *	8	0.0029 *	2	<0.0001 *
	High Exp.	86	27		19		29	

* statistically significant

**Table 4:** Multivariate analysis of DSS, LRFS, and MeFS in rectal cancer.

Parameter	DSS			LRFS			MeFS		
	H.R	95% CI	*p*-Value	H.R	95% CI	*p*-Value	H.R	95% CI	*p*-Value
Tumor regression grade	1.721	0.840–3.533	0.138	2.132	1.020–4.785	0.044 *	2.049	1.022–4.115	0.044 *
RGNEF expression	7.985	2.708–23.547	<0.001 *	2.235	0.899–5.558	0.084	17.011	3.986–72.602	<0.001 *
Vascular invasion	1.278	0.496–3.294	0.612	1.924	0.691–5.361	0.211	-	-	-
Post-Tx tumor status (ypT)	3.197	1.313–7.784	0.010 *	2.233	0.901–5.534	0.083	2.418	1.053–5.551	0.037 *
Pre-Tx nodal status (cN)	-	-	-	1.791	0.768–4.172	0.177	-	-	-

DSS, disease-specific survival; LRFS, local recurrence-free survival; MeFS, metastasis-free survival; * statistically significant.

## Data Availability

All data generated or analyzed during this study are included in this published article and its Supplementary Documentation File.

## Supplementary Materials

The following are available online at https://www.mdpi.com/article/10.3390/life12010018/s1, Table S1: The top 200 genes positively correlated with ARHGEF28, Table S2: The top 200 genes negatively correlated with ARHGEF28, Figure S1: The immunostaining of RGNEF and β-catenin, Figure S2: The correlation between ARHGEF28 and APC.
