# Supplementary material for: Utility of RGNEF in the Prediction of Clinical Prognosis in Patients with Rectal Cancer Receiving Preoperative Concurrent Chemoradiotherapy
Source: Life (Basel). 2021 Dec 23;12(1):18. doi: 10.3390/life12010018 (PMC8778573; doi:10.3390/life12010018)
Supplement: Supplementary file 1 [file life-12-00018-s001.zip › life-1485715-supplementary.pdf]

life

Supplementary Materials: Utility of RGNEF in the Prediction of Clinical Prognosis in Patients with Rectal Cancer Receiving Preoperative Concurrent Chemoradiotherapy

Table S1. The top 200 genes positively correlated with ARHGEF28.

Correlated Gene	Cytoband	Spearman's Correlation	p-Value	q-Value
MIA3	1q41	0.558	8.41×10^{-50}	1.67×10^{-45}
ABLIM1	10q25.3	0.544	5.67×10^{-47}	5.63×10^{-43}
KDM5B	1q32.1	0.529	5.52×10^{-44}	3.66×10^{-40}
MLPH	2q37.3	0.523	7.47×10^{-43}	3.71×10^{-39}
ARFGEF3	6q23.3-q24.1	0.505	1.39×10^{-39}	5.52×10^{-36}
ARHGAP26	5q31.3	0.496	4.38×10^{-38}	1.45×10^{-34}
TAOK3	12q24.23	0.495	7.11×10^{-38}	2.02×10^{-34}
TMTC2	12q21.31	0.487	1.52×10^{-36}	3.78×10^{-33}
PDE4D	5q11.2-q12.1	0.478	3.92×10^{-35}	8.65×10^{-32}
CCDC149	4p15.2	0.478	4.37×10^{-35}	8.67×10^{-32}
ARHGEF37	5q32	0.475	1.33×10^{-34}	2.40×10^{-31}
FRMD4B	3p14.1	0.474	1.47×10^{-34}	2.43×10^{-31}
KDM3B	5q31.2	0.472	3.92×10^{-34}	5.98×10^{-31}
MAPRE2	18q12.1-q12.2	0.47	6.28×10^{-34}	8.58×10^{-31}
ZBTB7C	18q21.1	0.47	6.48×10^{-34}	8.58×10^{-31}
PLD1	3q26.31	0.458	4.72×10^{-32}	5.86×10^{-29}
HID1	17q25.1	0.456	8.30×10^{-32}	9.70×10^{-29}
ASAP2	2p25.1|2p24	0.454	2.22×10^{-31}	2.45×10^{-28}
CREB3L1	11p11.2	0.451	6.04×10^{-31}	5.72×10^{-28}
MAST4	5q12.3	0.45	6.77×10^{-31}	6.11×10^{-28}
RASEF	9q21.32	0.448	1.24×10^{-30}	1.03×10^{-27}
GOLGB1	3q13.33	0.448	1.65×10^{-30}	1.31×10^{-27}
CBLB	3q13.11	0.447	2.15×10^{-30}	1.65×10^{-27}
RASA1	5q14.3	0.446	3.10×10^{-30}	2.28×10^{-27}
GCSAM	3q13.2	0.445	4.08×10^{-30}	2.89×10^{-27}
TRAK1	3p22.1	0.443	8.44×10^{-30}	5.79×10^{-27}
TOX	8q12.1	0.437	4.55×10^{-29}	2.83×10^{-26}
RAB27B	18q21.2	0.435	1.01×10^{-28}	5.91×10^{-26}
CAST	5q15	0.434	1.34×10^{-28}	7.61×10^{-26}
RABGAP1L	1q25.1	0.428	9.13×10^{-28}	4.77×10^{-25}
RNASEL	1q25.3	0.427	1.39×10^{-27}	7.09×10^{-25}
IQGAP2	5q13.3	0.424	3.03×10^{-27}	1.43×10^{-24}
SHROOM3	4q21.1	0.422	5.30×10^{-27}	2.45×10^{-24}
NECTIN4	1q23.3	0.422	6.41×10^{-27}	2.83×10^{-24}
MFSD6	2q32.2	0.418	1.81×10^{-26}	7.79×10^{-24}
GALNT10	5q33.2	0.418	1.84×10^{-26}	7.79×10^{-24}
PIK3C2B	1q32.1	0.416	3.57×10^{-26}	1.42×10^{-23}
FOXP1	3p13	0.416	4.01×10^{-26}	1.53×10^{-23}
SMARCA2	9p24.3	0.415	4.16×10^{-26}	1.56×10^{-23}
PTPN13	4q21.3	0.415	4.62×10^{-26}	1.70×10^{-23}
BHLHE41	12p12.1	0.415	5.53× 10-26	2.00× 10-23

Life **2022**, *12*, 18. https://doi.org/10.3390/life12010018 www.mdpi.com/journal/life

VWA3B	2q11.2	0.414	6.32× 10-26	2.20× 10-23
COL4A3BP	5q13.3	0.412	1.19× 10^{-25}	4.02× 10^{-23}
DOP1B	21q22.12	0.411	1.40× 10^{-25}	4.54× 10^{-23}
CARD6	5p13.1	0.411	1.52× 10^{-25}	4.87× 10^{-23}
AP3B1	5q14.1	0.411	1.67× 10^{-25}	5.26× 10^{-23}
ARHGAP21	10p12.1|10p12.3	0.41	2.14× 10^{-25}	6.65× 10^{-23}
DUSP4	8p12	0.409	2.52× 10^{-25}	7.70× 10^{-23}
SGMS2	4q25	0.409	2.75× 10^{-25}	8.29× 10^{-23}
AKAP13	15q25.3	0.409	2.95× 10^{-25}	8.74× 10^{-23}
CTNNA1	5q31.2	0.409	2.99× 10^{-25}	8.74× 10^{-23}
CACNA1C	12p13.33	0.409	3.14× 10^{-25}	8.91× 10^{-23}
MGLL	3q21.3	0.407	4.62× 10^{-25}	1.26× 10^{-22}
ERN2	16p12.2	0.407	4.73× 10^{-25}	1.27× 10^{-22}
PRUNE2	9q21.2	0.406	5.86× 10^{-25}	1.54× 10^{-22}
FGD4	12p11.21	0.406	5.88× 10^{-25}	1.54× 10^{-22}
PRRC1	5q23.2	0.406	7.64× 10^{-25}	1.97× 10^{-22}
DMXL1	5q23.1	0.405	8.55× 10^{-25}	2.18× 10^{-22}
EPHA4	2q36.1	0.405	9.33× 10^{-25}	2.35× 10^{-22}
CRYBG3	3q11.2	0.405	1.02× 10^{-24}	2.54× 10^{-22}
CEMIP2	9q21.13	0.402	2.43× 10^{-24}	5.74× 10^{-22}
GCC2	2q12.3	0.401	2.45× 10^{-24}	5.74× 10^{-22}
SWAP70	11p15.4	0.401	2.87× 10^{-24}	6.62× 10^{-22}
LINC00589	8p12	0.401	3.03× 10^{-24}	6.85× 10^{-22}
STXBP1	9q34.11	0.4	3.37× 10^{-24}	7.52× 10^{-22}
ATP8A1	4p13	0.4	3.79× 10^{-24}	8.36× 10^{-22}
HIPK2	7q34	0.398	6.52× 10^{-24}	1.41× 10^{-21}
CAMK2D	4q26	0.396	1.09× 10^{-23}	2.26× 10^{-21}
RASSF6	4q13.3	0.396	1.18× 10^{-23}	2.42× 10^{-21}
C3ORF52	3q13.2	0.396	1.20× 10^{-23}	2.42× 10^{-21}
MAP3K5	6q23.3	0.395	1.62× 10^{-23}	3.24× 10^{-21}
ANO1	11q13.3	0.395	1.64× 10^{-23}	3.27× 10^{-21}
ST5	11p15.4	0.393	2.32× 10^{-23}	4.48× 10^{-21}
APC	5q22.2	0.392	3.29× 10^{-23}	6.23× 10^{-21}
IKZF2	2q34	0.39	6.24× 10^{-23}	1.16× 10^{-20}
MARCH3	5q23.2	0.39	6.81× 10^{-23}	1.25× 10^{-20}
FGFR2	10q26.13	0.389	8.31× 10^{-23}	1.51× 10^{-20}
TIPARP	3q25.31	0.388	9.42× 10^{-23}	1.70× 10^{-20}
JAK1	1p31.3	0.388	9.76× 10^{-23}	1.73× 10^{-20}
CRACR2A	12p13.32	0.386	1.78× 10^{-22}	3.11× 10^{-20}
SLITRK6	13q31.1	0.385	2.09× 10^{-22}	3.61× 10^{-20}
ERGIC1	5q35.1	0.385	2.67× 10^{-22}	4.54× 10^{-20}
PDXDC1	16p13.11	0.384	3.01× 10^{-22}	5.06× 10^{-20}
EFNA5	5q21.3	0.384	3.03× 10^{-22}	5.06× 10^{-20}
STYK1	12p13.2	0.383	3.94× 10^{-22}	6.46× 10^{-20}
FER1L6	8q24.13	0.382	5.24× 10^{-22}	8.40× 10^{-20}
BAZ2B	2q24.2	0.382	5.63× 10^{-22}	8.88× 10^{-20}
GALNT4	12q21.33	0.381	6.20× 10^{-22}	9.47× 10^{-20}
LRRIQ4	3q26.2	0.381	6.32× 10^{-22}	9.58× 10^{-20}
PPFIBP1	12p11.23-p11.22	0.379	1.09× 10^{-21}	1.59× 10^{-19}
GAREM1	18q12.1	0.378	1.41× 10^{-21}	2.00× 10^{-19}

CDC42BPA	1q42.13	0.378	1.71×10^{-21}	2.41×10^{-19}
DST	6p12.1	0.377	1.81×10^{-21}	2.51×10^{-19}
MAML3	4q31.1	0.377	2.08×10^{-21}	2.83×10^{-19}
GNAQ	9q21.2	0.376	2.35×10^{-21}	3.15×10^{-19}
TLE4	9q21.31	0.376	2.40×10^{-21}	3.19×10^{-19}
LYST	1q42.3	0.376	2.50×10^{-21}	3.31×10^{-19}
FBXW11	5q35.1	0.376	2.71×10^{-21}	3.55×10^{-19}
RAP1GAP	1p36.12	0.376	2.76×10^{-21}	3.59×10^{-19}
AFAP1L2	10q25.3	0.375	3.32×10^{-21}	4.24×10^{-19}
TMEM131	2q11.2	0.375	3.35×10^{-21}	4.24×10^{-19}
SDR16C5	8q12.1	0.375	3.65×10^{-21}	4.59×10^{-19}
MARF1	16p13.11	0.374	4.48×10^{-21}	5.55×10^{-19}
AFF4	5q31.1	0.373	5.32×10^{-21}	6.52×10^{-19}
CYP3A5	7q22.1	0.373	5.36×10^{-21}	6.54×10^{-19}
IRF6	1q32.2	0.373	5.61×10^{-21}	6.80×10^{-19}
RAB5B	12q13.2	0.373	5.76×10^{-21}	6.94×10^{-19}
RASAL1	12q24.13	0.373	6.12×10^{-21}	7.28×10^{-19}
DNAH2	17p13.1	0.372	6.77×10^{-21}	8.01×10^{-19}
MARVELD2	5q13.2	0.372	6.89× 10-21	8.05× 10-19
LIMA1	12q13.12	0.372	7.37×10^{-21}	8.52×10^{-19}
TUT7	9q21.33	0.372	7.48×10^{-21}	8.59×10^{-19}
CD55	1q32.2	0.37	1.33×10^{-20}	1.47×10^{-18}
PPP3CA	4q24	0.369	1.48×10^{-20}	1.60×10^{-18}
ATP1B1	1q24.2	0.369	1.57× 10-20	1.69× 10-18
RUFY1	5q35.3	0.369	1.74×10^{-20}	1.85×10^{-18}
GOLGA2	9q34.11	0.368	1.78×10^{-20}	1.89×10^{-18}
GNE	9p13.3	0.367	2.34×10^{-20}	2.46×10^{-18}
WDR26	1q42.11-q42.12	0.367	2.38×10^{-20}	2.48×10^{-18}
CAPN8	1q41	0.367	2.57×10^{-20}	2.68×10^{-18}
STXBP5	6q24.3	0.367	2.65×10^{-20}	2.74×10^{-18}
TNIK	3q26.2-q26.31	0.367	2.86×10^{-20}	2.94×10^{-18}
VIT	2p22.2	0.365	4.07×10^{-20}	4.08×10^{-18}
KIAA0040	1q25.1	0.365	4.79×10^{-20}	4.74×10^{-18}
AHNAK	11q12.3	0.364	5.04×10^{-20}	4.96×10^{-18}
FKBP15	9q32	0.364	5.12×10^{-20}	5.02×10^{-18}
UAP1	1q23.3	0.364	5.52×10^{-20}	5.38×10^{-18}
SH3PXD2A	10q24.33	0.364	5.83×10^{-20}	5.62×10^{-18}
EDEM3	1q25.3	0.363	6.40×10^{-20}	6.11×10^{-18}
BACE2	21q22.2-q22.3	0.363	6.76×10^{-20}	6.40×10^{-18}
PLCL2	3p24.3	0.363	6.86×10^{-20}	6.46×10^{-18}
MAGI1	3p14.1	0.363	7.15×10^{-20}	6.67×10^{-18}
CADPS2	7q31.32	0.363	7.15×10^{-20}	6.67×10^{-18}
SP1	12q13.13	0.363	7.28×10^{-20}	6.76×10^{-18}
TBXAS1	7q34	0.363	7.60×10^{-20}	7.03×10^{-18}
TCN1	11q12.1	0.362	8.33×10^{-20}	7.62×10^{-18}
NCOA1	2p23.3	0.362	9.44×10^{-20}	8.49×10^{-18}
ABHD2	15q26.1	0.362	9.45×10^{-20}	8.49×10^{-18}
CKAP4	12q23.3	0.362	9.60×10^{-20}	8.56×10^{-18}
INPP1	2q32.2	0.361	1.08×10^{-19}	9.52×10^{-18}

ANKHD1-EIF4EBP3	5q31.3	0.36	1.32×10^{-19}	1.15×10^{-17}
VPS13D	1p36.22-p36.21	0.36	1.34×10^{-19}	1.17×10^{-17}
PROM1	4p15.32	0.36	1.48×10^{-19}	1.27×10^{-17}
FYCO1	3p21.31	0.36	1.56×10^{-19}	1.33×10^{-17}
OS9	12q13.3-q14.1	0.36	1.57×10^{-19}	1.33×10^{-17}
SMAD2	18q21.1	0.359	1.82×10^{-19}	1.53×10^{-17}
FBXO38	5q32	0.359	1.94×10^{-19}	1.61×10^{-17}
BCAS1	20q13.2	0.359	1.94×10^{-19}	1.61×10^{-17}
ACBD3	1q42.12	0.359	2.08×10^{-19}	1.72×10^{-17}
ATF7	12q13.13	0.359	2.14×10^{-19}	1.76×10^{-17}
ZSWIM6	5q12.1	0.359	2.15×10^{-19}	1.76×10^{-17}
RNF145	5q33.3	0.358	2.24×10^{-19}	1.83×10^{-17}
LPGAT1	1q32.3	0.358	2.31×10^{-19}	1.88×10^{-17}
GABRP	5q35.1	0.358	2.58×10^{-19}	2.09×10^{-17}
ARHGEF2	1q22	0.357	2.82×10^{-19}	2.28×10^{-17}
CAPN2	1q41	0.357	2.90×10^{-19}	2.32×10^{-17}
PIAS3	1q21.1	0.357	2.90×10^{-19}	2.32×10^{-17}
LINC00261	20p11.21	0.357	2.92×10^{-19}	2.33×10^{-17}
NSD1	5q35.3	0.357	3.02×10^{-19}	2.40×10^{-17}
CA8	8q12.1	0.357	3.35×10^{-19}	2.65×10^{-17}
SEC16A	9q34.3	0.356	3.76×10^{-19}	2.97×10^{-17}
PLCH1	3q25.31	0.356	4.25×10^{-19}	3.33×10^{-17}
DIP2B	12q13.12	0.356	4.46×10^{-19}	3.47×10^{-17}
MYRF	11q12.2	0.355	5.03×10^{-19}	3.88×10^{-17}
SPTSSB	3q26.1	0.355	5.24×10^{-19}	4.00×10^{-17}
FEM1C	5q22.3	0.355	5.32×10^{-19}	4.05×10^{-17}
KIF13B	8p12	0.354	5.75×10^{-19}	4.34×10^{-17}
ARHGEF12	11q23.3	0.354	6.79×10^{-19}	5.09×10^{-17}
PTGER2	14q22.1	0.354	7.02×10^{-19}	5.22×10^{-17}
SIDT1	3q13.2	0.354	7.22×10^{-19}	5.36×10^{-17}
AQP3	9p13.3	0.353	7.55×10^{-19}	5.57×10^{-17}
DYRK2	12q15	0.353	7.57×10^{-19}	5.57×10^{-17}
ARSJ	4q26	0.353	8.04×10^{-19}	5.89×10^{-17}
MYOF	10q23.33	0.353	8.13×10^{-19}	5.94×10^{-17}
LNX1	4q12	0.353	9.23×10^{-19}	6.69×10^{-17}
RPS6KA2	6q27	0.352	9.27×10^{-19}	6.70×10^{-17}
EFL1	15q25.2	0.352	9.33×10^{-19}	6.71×10^{-17}
RAPGEF3	12q13.11	0.352	1.15×10^{-18}	8.16×10^{-17}
MRAP2	6q14.2	0.351	1.18×10^{-18}	8.35×10^{-17}
LAMC2	1q25.3	0.351	1.27×10^{-18}	8.99×10^{-17}
TMEM246	9q31.1	0.35	1.67×10^{-18}	1.16×10^{-16}
AGBL4	1p33	0.35	1.79×10^{-18}	1.23×10^{-16}
LPIN2	18p11.31	0.349	1.98×10^{-18}	1.36×10^{-16}
IFT140	16p13.3	0.348	2.40×10^{-18}	1.63×10^{-16}
CCDC148	2q24.1	0.348	2.65×10^{-18}	1.79×10^{-16}
IFNE	9p21.3	0.348	2.69×10^{-18}	1.81×10^{-16}
SLK	10q24.33-q25.1	0.348	2.81×10^{-18}	1.89×10^{-16}
INSR	19p13.2	0.347	3.10×10^{-18}	2.07×10^{-16}
FAM114A1	4p14	0.347	3.15×10^{-18}	2.09×10^{-16}
TANC2	17q23.2-q23.3	0.347	3.15×10^{-18}	2.09×10^{-16}
F3	1p21.3	0.347	3.39×10^{-18}	2.23×10^{-16}

CHD9	16q12.2	0.347	3.48×10^{-18}	2.28×10^{-16}
TCP11L2	12q23.3	0.347	3.63×10^{-18}	2.38×10^{-16}
JADE2	5q31.1	0.346	4.07×10^{-18}	2.65×10^{-16}
IRF2	4q35.1	0.346	4.29×10^{-18}	2.79×10^{-16}
CYTH1	17q25.3	0.346	4.59×10^{-18}	2.96×10^{-16}
PLEKHG1	6q25.1	0.345	6.08×10^{-18}	3.88×10^{-16}
F11R	1q23.3	0.345	6.14×10^{-18}	3.89×10^{-16}
CNTRL	9q33.2	0.344	6.98×10^{-18}	4.40×10^{-16}
SCNN1A	12p13.31	0.344	7.28×10^{-18}	4.56×10^{-16}

Table S2. The top 200 genes negatively correlated with ARHGEF28.

Correlated Gene	Cytoband	Spearman's Correlation	p-Value	q-Value
PDRG1	20q11.21	−0.452	$4.03\times 10\times 10^{-31}$	4.22×10^{-28}
AHCY	20q11.22	−0.451	$5.35\times 10\times 10^{-31}$	5.32×10^{-28}
PIGU	20q11.22	−0.449	$9.84\times 10\times 10^{-31}$	8.50×10^{-28}
EIF6	20q11.22	−0.439	$2.64\times 10\times 10^{-29}$	1.74×10^{-26}
UBE2C	20q13.12	−0.439	$2.72\times 10\times 10^{-29}$	1.74×10^{-26}
PSMA7	20q13.33	−0.437	$5.43\times 10\times 10^{-29}$	3.27×10^{-26}
ROMO1	20q11.22	−0.433	$1.94\times 10\times 10^{-28}$	1.07×10^{-25}
MOCS3	20q13.13	−0.43	$4.81\times 10\times 10^{-28}$	2.58×10^{-25}
SUSD3	9q22.31	−0.425	$2.29\times 10\times 10^{-27}$	1.14×10^{-24}
TPD52L2	20q13.33	−0.424	$2.99\times 10\times 10^{-27}$	1.43×10^{-24}
DDX28	16q22.1	−0.422	$5.97\times 10\times 10^{-27}$	2.70×10^{-24}
SLC5A6	2p23.3	−0.418	$2.14\times 10\times 10^{-26}$	8.86×10^{-24}
SLC6A4	17q11.2	−0.417	$2.71\times 10\times 10^{-26}$	1.10×10^{-23}
SNTA1	20q11.21	−0.416	$3.81\times 10\times 10^{-26}$	1.48×10^{-23}
RANBP1	22q11.21	−0.414	$5.77\times 10\times 10^{-26}$	2.05×10^{-23}
MRGBP	20q13.33	−0.413	$8.28\times 10\times 10^{-26}$	2.84×10^{-23}
RASL10B	17q12	−0.411	$1.37\times 10\times 10^{-25}$	4.54×10^{-23}
ARL2	11q13.1	−0.409	$3.07\times 10\times 10^{-25}$	8.83×10^{-23}
FCGRT	19q13.33	−0.408	$3.53\times 10\times 10^{-25}$	9.87×10^{-23}

SAC3D1	11q13.1	−0.407	$4.51\times 10\times 10^{-25}$	1.25×10^{-22}
TP53RK	20q13.12	−0.404	$1.16\times 10\times 10^{-24}$	2.85×10^{-22}
PLA2G12B	10q22.1	−0.404	$1.27\times 10\times 10^{-24}$	3.08×10^{-22}
ADRM1	20q13.33	−0.403	$1.82\times 10\times 10^{-24}$	4.35×10^{-22}
HM13	20q11.21	−0.401	$2.92\times 10\times 10^{-24}$	6.66×10^{-22}
RAE1	20q13.31	−0.399	$4.36\times 10\times 10^{-24}$	9.53×10^{-22}
BYSL	6p21.1	−0.397	$7.99\times 10\times 10^{-24}$	1.71×10^{-21}
RALY	20q11.22	−0.397	$9.12\times 10\times 10^{-24}$	1.93×10^{-21}
TOMM34	20q13.12	−0.397	$9.45\times 10\times 10^{-24}$	1.98×10^{-21}
PYCR3	8q24.3	−0.394	$2.19\times 10\times 10^{-23}$	4.31×10^{-21}
MYBL2	20q13.12	−0.394	$2.26\times 10\times 10^{-23}$	4.41×10^{-21}
CCDC167	6p21.2	−0.393	$2.81\times 10\times 10^{-23}$	$5.37\times 10^{-2}1$
RPS21	20q13.33	−0.392	$3.94\times 10\times 10^{-23}$	7.39×10^{-21}
SNRPC	6p21.31	−0.388	$9.56\times 10\times 10^{-23}$	1.71×10^{-20}
SLC37A4	11q23.3	−0.387	$1.54\times 10\times 10^{-22}$	2.70×10^{-20}
POLR2G	11q12.3	−0.385	$2.29\times 10\times 10^{-22}$	3.92×10^{-20}
SMTNL2	17p13.2	−0.384	$3.08\times 10\times 10^{-22}$	5.09×10^{-20}
SLC30A2	1p36.11	−0.382	$4.72\times 10\times 10^{-22}$	7.69×10^{-20}
EIF2S2	20q11.22	−0.382	$5.07\times 10\times 10^{-22}$	8.20×10^{-20}
CSTF1	20q13.2-q13.31	−0.382	$5.58\times 10\times 10^{-22}$	8.86×10^{-20}
POLDIP2	17q11.2	−0.382	$5.78\times 10\times 10^{-22}$	9.03×10^{-20}
EEF2KMT	16p13.3	−0.382	$5.92\times 10\times 10^{-22}$	9.20×10^{-20}
MRPL49	11q13.1	−0.381	$6.13\times 10\times 10^{-22}$	9.44×10^{-20}
NELFCD	20q13.32	−0.381	$6.42\times 10\times 10^{-22}$	9.67×10^{-20}
SCAND1	20q11.23	−0.38	$8.60\times 10\times 10^{-22}$	1.29×10^{-19}
FITM2	20q13.12	−0.38	$9.33\times 10\times$	1.38×10^{-19}

			10^{-22}	
PKLR	1q22	−0.379	1.04× 10× 10^{-21}	1.53× 10^{-19}
KHK	2p23.3	−0.379	1.12× 10× 10^{-21}	1.63× 10^{-19}
E2F1	20q11.22	−0.379	1.15× 10× 10^{-21}	1.66× 10^{-19}
TIMM50	19q13.2	−0.378	1.37× 10× 10^{-21}	1.96× 10^{-19}
CELP	9q34.13	−0.377	1.74× 10× 10^{-21}	2.44× 10^{-19}
ABT1	6p22.2	−0.377	1.88× 10× 10^{-21}	2.60× 10^{-19}
TRMT61A	14q32.33	−0.377	1.91× 10× 10^{-21}	2.61× 10^{-19}
CDK5RAP1	20q11.21	−0.376	2.26× 10× 10^{-21}	3.06× 10^{-19}
PDF	16q22.1	−0.376	2.60× 10× 10^{-21}	3.42× 10^{-19}
GAL	11q13.2	−0.375	3.19× 10× 10^{-21}	4.11× 10^{-19}
OIT3	10q22.1	−0.375	3.34× 10× 10^{-21}	4.24× 10^{-19}
BCS1L	2q35	−0.374	4.06× 10× 10^{-21}	5.07× 10^{-19}
EPOP	17q12	−0.374	4.50× 10× 10^{-21}	5.55× 10^{-19}
MRPL11	11q13.2	−0.373	5.99× 10× 10^{-21}	7.17× 10^{-19}
TMEM223	11q12.3	−0.372	6.87× 10× 10^{-21}	8.05× 10^{-19}
ADSL	22q13.1	−0.372	7.06× 10× 10^{-21}	8.20× 10^{-19}
ACOT8	20q13.12	−0.372	7.74× 10× 10^{-21}	8.84× 10^{-19}
CTU2	16q24.3	−0.371	8.23× 10× 10^{-21}	9.34× 10^{-19}
CCDC86	11q12.2	−0.371	8.47× 10× 10^{-21}	9.56× 10^{-19}
AURKA	20q13.2	−0.371	8.67× 10× 10^{-21}	9.74× 10^{-19}
POLR2D	2q14.3	−0.371	8.85× 10× 10^{-21}	9.88× 10^{-19}
TIMM17B	Xp11.23	−0.371	1.02× 10× 10^{-20}	1.14× 10^{-18}
SELENOH	11q12.1	−0.369	1.39× 10× 10^{-20}	1.51× 10^{-18}
QPRT	16p11.2	−0.369	1.39× 10× 10^{-20}	1.51× 10^{-18}
YDJC	22q11.21	−0.369	1.44× 10× 10^{-20}	1.56× 10^{-18}

LRP3	19q13.11	−0.367	$2.33\times 10\times 10^{-20}$	2.46×10^{-18}
ERAL1	17q11.2	−0.366	$3.24\times 10\times 10^{-20}$	3.31×10^{-18}
SLC25A39	17q21.31	−0.366	$3.33\times 10\times 10^{-20}$	3.39×10^{-18}
THOC6	16p13.3	−0.366	$3.41\times 10\times 10^{-20}$	3.46×10^{-18}
HGH1	8q24.3	−0.365	$3.85\times 10\times 10^{-20}$	3.89×10^{-18}
PDXP	22q13.1	−0.365	$4.10\times 10\times 10^{-20}$	4.10×10^{-18}
EREG	4q13.3	−0.365	$4.23\times 10\times 10^{-20}$	4.20×10^{-18}
DDN	12q13.12	−0.364	$5.81\times 10\times 10^{-20}$	5.62×10^{-18}
LINC01816	2p13.3	−0.363	$6.34\times 10\times 10^{-20}$	6.09×10^{-18}
CSE1L	20q13.13	−0.363	$6.74\times 10\times 10^{-20}$	6.40×10^{-18}
TRMT112	11q13.1	−0.362	$8.26\times 10\times 10^{-20}$	7.60×10^{-18}
G6PC3	17q21.31	−0.362	$8.63\times 10\times 10^{-20}$	7.86×10^{-18}
UCKL1	20q13.33	−0.362	$9.16\times 10\times 10^{-20}$	8.31×10^{-18}
EMC8	16q24.1	−0.362	$9.49\times 10\times 10^{-20}$	8.49×10^{-18}
POLR2F	22q13.1	−0.362	$1.01\times 10\times 10^{-19}$	8.98×10^{-18}
GSS	20q11.22	−0.361	$1.16\times 10\times 10^{-19}$	1.02×10^{-17}
DTYMK	2q37.3	−0.36	$1.36\times 10\times 10^{-19}$	1.18×10^{-17}
SLC3A2	11q12.3	−0.36	$1.38\times 10\times 10^{-19}$	1.20×10^{-17}
UQCC1	20q11.22	−0.36	$1.47\times 10\times 10^{-19}$	1.26×10^{-17}
HTRA2	2p13.1	−0.36	$1.67\times 10\times 10^{-19}$	1.41×10^{-17}
UQCC3	11q12.3	−0.359	$1.90\times 10\times 10^{-19}$	1.59×10^{-17}
DDT	22q11.23	−0.356	$3.97\times 10\times 10^{-19}$	3.11×10^{-17}
TLCD1	17q11.2	−0.355	$4.66\times 10\times 10^{-19}$	3.61×10^{-17}
NUTF2	16q22.1	−0.355	$4.66\times 10\times 10^{-19}$	3.61×10^{-17}
POFUT1	20q11.21	−0.355	$5.18\times 10\times 10^{-19}$	3.98×10^{-17}
ACTR5	20q11.23	−0.355	$5.43\times 10\times$	4.12×10^{-17}

			10^{-19}	
PM20D2	6q15	−0.354	6.50× 10× 10^{-19}	4.89× 10^{-17}
C8ORF33	8q24.3	−0.354	6.86× 10× 10^{-19}	5.12× 10^{-17}
PNO1	2p14	−0.353	8.80× 10× 10^{-19}	6.40× 10^{-17}
GPR143	Xp22.2	−0.352	1.08× 10× 10^{-18}	7.73× 10^{-17}
ACTR3C	7q36.1	−0.352	1.09× 10× 10^{-18}	7.81× 10^{-17}
WDR74	11q12.3	−0.351	1.29× 10× 10^{-18}	9.09× 10^{-17}
PIPOX	17q11.2	−0.351	1.35× 10× 10^{-18}	9.46× 10^{-17}
SYS1	20q13.12	−0.35	1.51× 10× 10^{-18}	1.06× 10^{-16}
FAM86C1	11q13.4	−0.35	1.65× 10× 10^{-18}	1.15× 10^{-16}
MCUR1	6p23	−0.35	1.74× 10× 10^{-18}	1.21× 10^{-16}
ALYREF	17q25.3	−0.35	1.86× 10× 10^{-18}	1.28× 10^{-16}
DUSP15	20q11.21	−0.349	2.17× 10× 10^{-18}	1.48× 10^{-16}
SLC22A11	11q13.1	−0.348	2.62× 10× 10^{-18}	1.78× 10^{-16}
SSBP4	19p13.11	−0.348	2.82× 10× 10^{-18}	1.89× 10^{-16}
FADS3	11q12.2	−0.347	3.38× 10× 10^{-18}	2.23× 10^{-16}
LAMTOR1	11q13.4	−0.346	4.34× 10× 10^{-18}	2.81× 10^{-16}
NAALADL1	11q13.1	−0.345	4.91× 10× 10^{-18}	3.16× 10^{-16}
GPS1	17q25.3	−0.345	5.04× 10× 10^{-18}	3.23× 10^{-16}
ZSWIM1	20q13.12	−0.345	6.10× 10× 10^{-18}	3.88× 10^{-16}
ASCL2	11p15.5	−0.344	6.34× 10× 10^{-18}	4.01× 10^{-16}
GCSH	16q23.2	−0.344	7.17× 10× 10^{-18}	4.51× 10^{-16}
DPM1	20q13.13	−0.342	1.01× 10× 10^{-17}	6.15× 10^{-16}
AUP1	2p13.1	−0.342	1.04× 10× 10^{-17}	6.28× 10^{-16}
EDEM2	20q11.22	−0.342	1.07× 10× 10^{-17}	6.42× 10^{-16}
RAB5IF	20q11.23	−0.342	1.10× 10× 10^{-17}	6.59× 10^{-16}

CHCHD2	7p11.2	−0.342	1.10× 10× 10^{-17}	6.59× 10^{-16}
COA4	11q13.4	−0.342	1.11× 10× 10^{-17}	6.59× 10^{-16}
NECAB3	20q11.22	−0.341	1.35× 10× 10^{-17}	7.89× 10^{-16}
DNAJA3	16p13.3	−0.341	1.35× 10× 10^{-17}	7.89× 10^{-16}
EXOSC4	8q24.3	−0.341	1.35× 10× 10^{-17}	7.89× 10^{-16}
AAR2	20q11.23	−0.341	1.41× 10× 10^{-17}	8.20× 10^{-16}
PSMG1	21q22.2	−0.341	1.55× 10× 10^{-17}	8.93× 10^{-16}
PISD	22q12.2	−0.34	1.67× 10× 10^{-17}	9.64× 10^{-16}
PROCR	20q11.22	−0.34	1.81× 10× 10^{-17}	1.04× 10^{-15}
DRG1	22q12.2	−0.34	1.91× 10× 10^{-17}	1.09× 10^{-15}
PAAF1	11q13.4	−0.339	2.11× 10× 10^{-17}	1.19× 10^{-15}
CPNE1	20q11.22	−0.338	2.46× 10× 10^{-17}	1.38× 10^{-15}
SCARNA12	12p13.31	−0.338	2.55× 10× 10^{-17}	1.42× 10^{-15}
MRPS23	17q22	−0.338	2.75× 10× 10^{-17}	1.52× 10^{-15}
MRPL2	6p21.1	−0.338	2.95× 10× 10^{-17}	1.61× 10^{-15}
TYSND1	10q22.1	−0.338	3.03× 10× 10^{-17}	1.65× 10^{-15}
GZMB	14q12	−0.336	3.88× 10× 10^{-17}	2.08× 10^{-15}
SNHG11	20q11.23	−0.336	4.03× 10× 10^{-17}	2.15× 10^{-15}
MRPL38	17q25.1	−0.336	4.80× 10× 10^{-17}	2.55× 10^{-15}
CKMT2	5q14.1	−0.335	5.27× 10× 10^{-17}	2.79× 10^{-15}
RPN2	20q11.23	−0.335	5.30× 10× 10^{-17}	2.79× 10^{-15}
TOMM22	22q13.1	−0.335	5.32× 10× 10^{-17}	2.79× 10^{-15}
CHST13	3q21.3	−0.335	5.33× 10× 10^{-17}	2.79× 10^{-15}
DCUN1D5	11q22.3	−0.335	5.59× 10× 10^{-17}	2.91× 10^{-15}
TTI1	20q11.23	−0.334	6.58× 10× 10^{-17}	3.39× 10^{-15}
GRINA	8q24.3	−0.334	7.12× 10×	3.66× 10^{-15}

			10^{-17}	
CLDN9	16p13.3	−0.333	7.79× 10× 10^{-17}	3.98× 10^{-15}
CHKB-DT	22q13.33	−0.333	8.04× 10× 10^{-17}	4.08× 10^{-15}
TRMU	22q13.31	−0.333	8.22× 10× 10^{-17}	4.14× 10^{-15}
MYC	8q24.21	−0.333	8.25× 10× 10^{-17}	4.15× 10^{-15}
CCDC85B	11q13.1	−0.332	9.64× 10× 10^{-17}	4.79× 10^{-15}
NME1	17q21.33	−0.332	9.78× 10× 10^{-17}	4.85× 10^{-15}
C6ORF120	6q27	−0.332	9.96× 10× 10^{-17}	4.92× 10^{-15}
OSER1	20q13.12	−0.332	1.01× 10× 10^{-16}	4.98× 10^{-15}
MED30	8q24.11	−0.332	1.01× 10× 10^{-16}	4.99× 10^{-15}
XYLB	3p22.2	−0.332	1.08× 10× 10^{-16}	5.30× 10^{-15}
ATP5F1EP2	13q12.2	−0.332	1.10× 10× 10^{-16}	5.36× 10^{-15}
OTUB1	11q13.1	−0.332	1.11× 10× 10^{-16}	5.40× 10^{-15}
MAIP1	2q33.1	−0.332	1.14× 10× 10^{-16}	5.52× 10^{-15}
SPINDOC	11q13.1	−0.331	1.49× 10× 10^{-16}	7.15× 10^{-15}
E2F4	16q22.1	−0.33	1.51× 10× 10^{-16}	7.24× 10^{-15}
CD81	11p15.5	−0.33	1.54× 10× 10^{-16}	7.33× 10^{-15}
NEU1	6p21.33	−0.33	1.68× 10× 10^{-16}	8.00× 10^{-15}
SP6	17q21.32	−0.33	1.69× 10× 10^{-16}	8.02× 10^{-15}
RPS6KB2	11q13.2	−0.33	1.85× 10× 10^{-16}	8.69× 10^{-15}
VWCE	11q12.2	−0.329	2.04× 10× 10^{-16}	9.55× 10^{-15}
NIP7	16q22.1	−0.329	2.15× 10× 10^{-16}	1.00× 10^{-14}
AGMAT	1p36.21	−0.329	2.19× 10× 10^{-16}	1.02× 10^{-14}
DYNLRB1	20q11.22	−0.328	2.51× 10× 10^{-16}	1.15× 10^{-14}
SLC2A8	9q33.3	−0.328	2.86× 10× 10^{-16}	1.30× 10^{-14}
ECE2	3q27.1	−0.326	3.60× 10× 10^{-16}	1.61× 10^{-14}

PEPD	19q13.11	−0.326	3.75× 10× 10^{-16}	1.67× 10^{-14}
PPP1CA	11q13.2	−0.326	3.77× 10× 10^{-16}	1.68× 10^{-14}
SLC39A5	12q13.3	−0.326	3.81× 10× 10^{-16}	1.69× 10^{-14}
MANBAL	20q11.23	−0.326	4.27× 10× 10^{-16}	1.89× 10^{-14}
SRSF2	17q25.1	−0.325	4.86× 10× 10^{-16}	2.14× 10^{-14}
C16ORF91	16p13.3	−0.325	5.15× 10× 10^{-16}	2.25× 10^{-14}
NAA10	Xq28	−0.324	5.67× 10× 10^{-16}	2.46× 10^{-14}
TBRG4	7p13	−0.324	5.92× 10× 10^{-16}	2.55× 10^{-14}
PRDX4	Xp22.11	−0.324	5.96× 10× 10^{-16}	2.56× 10^{-14}
H2AFX	11q23.3	−0.324	6.23× 10× 10^{-16}	2.67× 10^{-14}
HDHD5	22q11.1	−0.324	6.30× 10× 10^{-16}	2.69× 10^{-14}
FAH	15q25.1	−0.324	6.48× 10× 10^{-16}	2.75× 10^{-14}
CISD3	17q12	−0.324	6.55× 10× 10^{-16}	2.77× 10^{-14}
YTHDF1	20q13.33	−0.324	6.88× 10× 10^{-16}	2.89× 10^{-14}
EXOSC5	19q13.2	−0.323	7.42× 10× 10^{-16}	3.10× 10^{-14}
NSUN5	7q11.23	−0.323	7.47× 10× 10^{-16}	3.11× 10^{-14}
PLAGL2	20q11.21	−0.323	7.49× 10× 10^{-16}	3.11× 10^{-14}
TOMM40	19q13.32	−0.323	7.73× 10× 10^{-16}	3.21× 10^{-14}
UTP4	16q22.1	−0.323	7.84× 10× 10^{-16}	3.25× 10^{-14}
CEL	9q34.13	−0.323	8.18× 10× 10^{-16}	3.38× 10^{-14}
RPP40	6p25.1	−0.322	8.67× 10× 10^{-16}	3.57× 10^{-14}
SNAI1	20q13.13	−0.322	9.87× 10× 10^{-16}	4.03× 10^{-14}
GADD45GIP1	19p13.13	−0.321	1.11× 10× 10^{-15}	4.54× 10^{-14}
CXCR3	Xq13.1	−0.321	1.21× 10× 10^{-15}	4.91× 10^{-14}
SLC25A19	17q25.1	−0.321	1.22× 10× 10^{-15}	4.93× 10^{-14}
NFS1	20q11.22	−0.321	1.29× 10×	5.18× 10^{-14}

			10^{-15}	
EMG1	12p13.31	−0.321	1.30× 10× 10^{-15}	5.23× 10^{-14}
RPUSD1	16p13.3	−0.321	1.30× 10× 10^{-15}	5.23× 10^{-14}

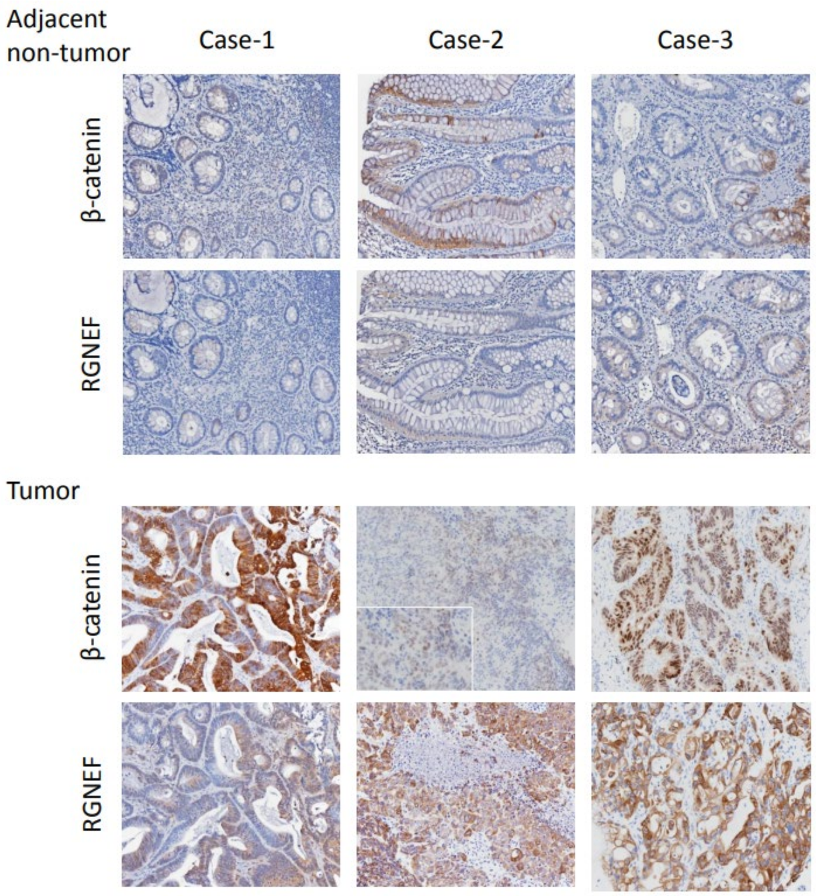

Figure S1: The immunostaining of RGNEF and β-catenin.

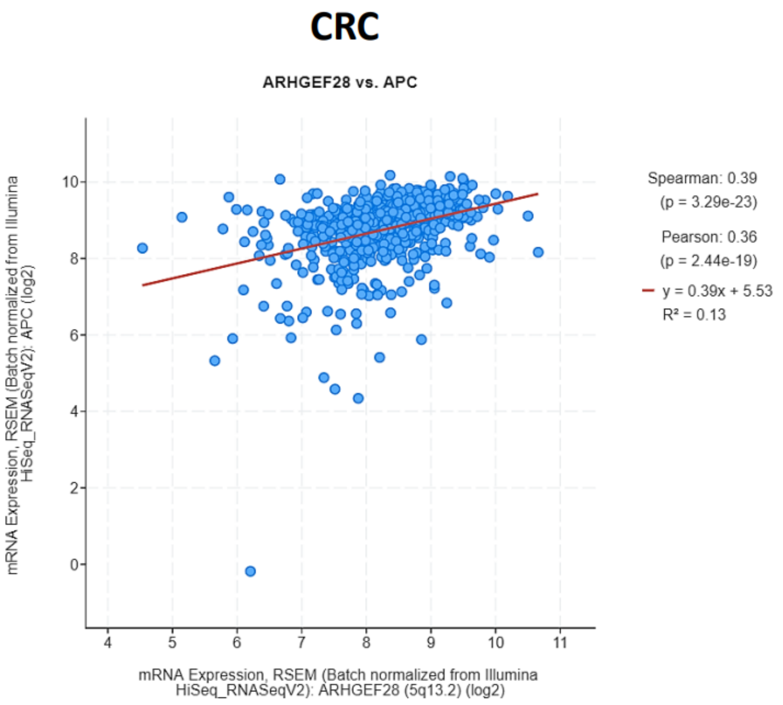

Figure S2: The correlation between ARHGEF28 and APC.
